# Revolutionizing bone regeneration and wound healing: Mechanical stromal vascular fraction and hyaluronic acid in a mouse calvarial defect model

**DOI:** 10.3389/fcell.2025.1582083

**Published:** 2025-05-13

**Authors:** Riccardo Ossanna, Lindsey Alejandra Quintero Sierra, Sara Ghazanfar Tehrani, Vivekanand Jha, Chiara Curatola, Alice Busato, Anita Conti, Giamaica Conti, Nicola Zingaretti, Pier Camillo Parodi, Francesco De Francesco, Michele Riccio, Andrea Sbarbati

**Affiliations:** ^1^ Department of Neuroscience, Biomedicine, and Movement, Section of Anatomy and Histology, University of Verona, Verona, Italy; ^2^ Aptuit and Evotec Company, Safety and Assessment Department, Verona, Italy; ^3^ Clinic of Plastic and Reconstructive Surgery, Academic Hospital of Udine, Department of Medical Area (DAME), University of Udine, Udine, Italy; ^4^ Department of General and Specialties Surgery, SOD of Reconstructive Surgery and Hand Surgery, Azienda Ospedaliera Universitaria Delle Marche, Ancona, Italy

**Keywords:** bone repair, stromal vascular fraction, calvarial bone defects, hyaluronic acid, adipose-derived stem cell

## Abstract

**Introduction:**

The stromal vascular fraction (SVF) is a complex and heterogeneous suspension derived from adipose tissue, containing both cellular and noncellular components. Its cellular fraction includes adipose-derived stem cells (ASCs), endothelial precursor cells, pericytes, macrophages, lymphocytes, and smooth muscle cells. The acellular “secretome” of SVF includes bioactive molecules such as growth factors, cytokines, chemokines, extracellular vesicles, and fragments of extracellular matrix (ECM), which contribute to its regenerative potential. Bone defeatures can be stimulated by mesenchymal stem cells (MSCs) that differentiate into osteoblast to support the healing and repair process. In addition to its cell content, the SVF is rich in growth factors, cytokines and chemokines, extracellular vesicles, and extracellular matrix components, which could stimulate regenerative processes through a trophic effect. Studies showed that hyaluronic acids are usually involved in healing processes. This study was focused on the healing potency of stromal stem cells isolated from adipose tissues by mechanical digestion, and the role of low-molecular-weight hyaluronic acid (LMW-HA, ACP) in the healing process was tested in calvarial defeatures in a mouse model, in comparison with the enzymatic digestion method.

**Methods:**

The bone healing and remodeling process was evaluated *in vivo* using magnetic resonance imaging (MRI) up to 15 days post-treatment, and differences in the quality of bone regeneration were assessed by *ex vivo* histological analysis, immunofluorescences, and ultrastructural analysis. The bone matrix formed after treatment with mechanically digested Hy tissue stromal vascular fraction + hyaluronic acid (HT-SVF + ACP) was compared to that formed with enzymatically digested stromal vascular fraction + hyaluronic acid (ED-SVF + ACP), with the saline group serving as the control group.

**Results:**

In this study, we explore a groundbreaking approach using HT-SVF combined with ACP to promote bone regeneration. Through comparative analysis with ED-SVF in a calvarial defect mouse model, we demonstrate the superior efficacy of HT-SVF + ACP in enhancing bone healing, reducing fibrotic tissue, and improving bone matrix maturity.

**Discussion:**

The findings establish the potential of HT-SVF as a cost-effective and efficient method for bone regenerative therapy.

## Introduction

Bone is a dynamic tissue with a highly vascularized dense structure that enables its unique capacity to leave no scars after the healing and remodeling processes (Simunovic and Finkenzeller, 2021). The stress and biological environment are relevant to bone function and structure formation (Rubin et al., 2001). New bone development is thought to occur under low oxygen tension (Brighton and Krebs, 1972), allowing the expression of specific genes that increase cell survival under hypoxic conditions and re-establish the vasculature for oxygen delivery (Semenza, 2000). In addition, hypoxia induces the production of chemotactic factors implicated in cell migration, differentiation, and new bone formation.

During the natural healing process of bone, the reparative cascade is activated. Platelets, inflammatory cells, and stem cells arrive at the site of injury, secreting cytokines and growth factors, including IL-1 to IL-6, platelet-derived growth factor (PDGF), vascular endothelial growth factor (VEGF), and bone morphogenetic protein (BMP) (Rui et al., 2012). This cellular response leads to the invasion of mesenchymal stem cells (MSCs), which differentiate into osteoblasts and chondrocytes to complete the repair (Schindeler et al., 2008). Numerous studies have confirmed that MSCs form bone by differentiating into osteoblasts (Bruder et al., 1998; Gu et al., 2012; Zhu et al., 2012). In addition, many other studies have confirmed that the recruitment of factors with an adequate amount of MSCs and the micro-environment around the fracture are effective for fracture repair (Tsai et al., 2012; Zhi et al., 2011; Zuo et al., 2013).

Bone defects often arise from trauma, tumor resection, reconstructive surgery, congenital malformations, infections, and orthopedic or maxillofacial surgery procedures, and promoting bone regeneration is a unique challenge for both clinicians and scientists. Based on these observations, the regenerative medicine could be considered one of the major promises for all the patients suffering from bone problems, which has a huge detrimental effect on patient’s lives and society (Majidinia et al., 2018). Unlike in other tissues, most bone injuries (fractures) heal without forming scar tissue, and bone is regenerated with its preexisting properties (Einhorn, 1998). However, there are complex clinical conditions in which a large amount of bone regeneration is required, such as skeletal reconstruction of extensive bone defects created by trauma, infection, tumor resection, skeletal abnormalities, or cases in which the regenerative process is compromised, such as vascular necrosis and osteoporosis (Dimitriou et al., 2011). In fields such as oral and maxillofacial surgery, several treatment methods are currently available, which can be used either alone or in combination for the enhancement or management of these complex clinical situations (Dimitriou et al., 2011). Standard approaches clinically applied to stimulate or augment bone regeneration included distraction osteogenesis and bone transplantation (Aronson, 1997; Green et al., 1992), but there are also other several bone-grafting methods, such as autologous bone grafts, allografts, and bone-graft substitutes or growth factors (Giannoudis et al., 2005; Giannoudis and Einhorn, 2009). Furthermore, bone grafting is commonly performed in orthopedic and maxillofacial surgery as a “gold standard” approach (Bauer and Muschler, 2000) because it is the patient’s own tissue; autologous bone is histocompatible and non-immunogenic, reducing the likelihood of immunoreactions and transmission of infections to a minimum. Nevertheless, harvesting requires an additional surgical procedure, with well-documented complications and discomfort for the patient (Ahlmann et al., 2002). In this direction, wide progress has been made in cellular and molecular biology, allowing detailed histological analyses, *in vitro* and *in vivo* characterization of bone-forming cells, and identification of transcriptional and translational profiles of the genes and proteins involved in the process of bone regeneration and fracture repair (Komatsu and Warden, 2010). With an improved understanding of fracture healing and bone regeneration at the molecular level (Dimitriou et al., 2005), a number of key molecules that regulate this complex physiological process have been identified and are already in clinical use or under investigation to enhance bone repair. Among these molecules, bone morphogenic proteins (BMPs) have been the most extensively studied as they are potent osteo-inductive factors.

However, there are several issues with their use, including safety (because of the supraphysiological concentrations of growth factors needed to obtain the desired osteo-inductive effects), the high cost of treatment, and, more importantly, the potential for ectopic bone formation (Argintar et al., 2011).

MSCs or stromal stem cells are adult cells isolated from mesenchymal tissues. They have the property of being able to differentiate into many other cell types (Quintero Sierra et al., 2023; Wakao et al., 2011; Kuroda et al., 2013). The application of stem cells in cell-based therapy promotes the repair response of diseased, dysfunctional, or injured tissue. This is obtained by using both the stem cell progenitor, which *in vitro* differentiates into tissue-specific cells, and their derivative (a subpopulation of mature cells derived from stem cell differentiation *in vitro*) (Kuroda et al., 2013). In humans, MSC sources include bone marrow, adipose tissue, umbilical cord tissue, dermis, and peripheral blood (Sato et al., 2020). The fact that these MSCs are adipose tissue-resident cells makes them potentially attractive as they could be extracted and isolated from easily harvested fat by liposuction (Wakao et al., 2011; Wakao et al., 2014; Dai Prè et al., 2020). The application of MSCs in regenerative medicine presents two key advantages: first, a low risk of tumorigenesis, and second, a high differentiation capacity, which permits their application to the restoration of various pathological cell loss conditions (Wakao et al., 2011; Wakao et al., 2014; Rigotti et al., 2009; Heneidi et al., 2013).

Moreover, due to their origin from mesodermal lineage, they are able to differentiate into cells such as osteocytes, chondrocytes, adipocytes, skeletal muscle cells, endothelial cells, and cardiomyocytes. Therefore, they can find numerous applications in regenerative therapy studies (De Francesco et al., 2021a; Ogura et al., 2014; Ossanna et al., 2024).

Another alternative cellular source is the stromal vascular fraction (SVF). The SVF consists of adipose-derived stem cells (ASCs), endothelial precursor cells, macrophages, smooth muscle cells, lymphocytes, pericytes, and pre-adipocytes (Grünherz et al., 2019; Lo Furno et al., 2017; Capuzzo et al., 2023). Moreover, the SVF secretum consists of proliferative, pro-angiogenic, pro-differentiative, and pro-antiapoptotic factors, along with extracellular matrix components (Quintero Sierra et al., 2023; Simonacci et al., 2017; De Francesco et al., 2021b; Kachgal and Putnam, 2011). Altogether, the SVF contains a milieu of cells that constitute the functional cellular niche for regenerative processes (Xue et al., 2020; Rosa et al., 2021). Interestingly, adipose tissue contains a higher number of stem cells than bone marrow (Doornaert et al., 2019), making it a more effective source (500 times greater when counted per unit volume than bone marrow) with no ethical concerns regarding its procurement (Deptuła et al., 2021). The ASC content within the SVF of lipoaspirate samples is well known, and consequently, the SVF is used in several therapies and in a wide range of human regenerative medicine approaches (De Francesco et al., 2024). The contents of SVF can promote revascularization, activate local stem cell niches, modulate immune responses *via* paracrine secretion of numerous bioactive molecules, promote wound healing, exhibit anti-inflammatory activity, and promote angiogenesis. These regenerative effects could be partially due to the MSC content of the SVF (Capuzzo et al., 2023; Bora and Majumdar, 2017; Luck et al., 2018).

The SVF can be obtained from adipose tissue either through enzymatic methods by using collagenase enzyme that allows to separation the contents into two distinct phases: the floating mature adipocyte fraction and the cellular components in the lower aqueous fraction (Matsumoto et al., 2006; Zuk et al., 2001), resulting in an MSC-rich product, or non-enzymatic techniques, in which their mechanical strategies help desegregate adipose tissue, with cellular yield lower than enzymatic methods but with highly preserved micro-fragments of connective tissue (Quintero Sierra et al., 2023). Altogether, the SVF contains a milieu of cells that constitute the functional cellular niche for regenerative processes (De Francesco et al., 2009; Ferraro et al., 2013).

Hyaluronic acid, a critical component of the extracellular matrix, is a good example of natural molecule that has been applied in regenerative medicine with good results (Valachová and Šoltés, 2021; Carton and Malatesta, 2024). In the last decade, it has become clear that, in addition to their scaffolding properties, low-molecular-weight hyaluronic acid (LMW-HA) molecules can also contribute to other mechanisms like in the processing of injury and healing (Marinho et al., 2021).

In this work, we aim to evaluate the regenerative potential of SVF, containing the stem cell niches activated by mechanical processes (HT-SVF) and mixed with a low molecular weight hyaluronic acid (ACP) as a scaffold, in order to maintain the pro-regenerative product in the calvarial defect locus during the reparative process, and comparing the obtained product by enzymatic digestion mixed with ACP, along with control groups (ACP and saline). In particular, our goal was to investigate the ability of wound healing and tissue repairing, specifically in the case of hard tissues like bone, which was promoted by a new mechanical stromal vascular fraction in a calvarial defect model.

## Materials and methods

### Fat harvesting

The adipose tissue was harvested from women (n = 3) subjected to ambulatory liposuction, aged between 41 and 69 years. Informed consent was signed by patients prior to tissue harvesting, and all processes were carried out according to the ethical guidelines established by the review committee for human studies of AOU “delle Marche,” Ancona, Italy (Micro-adipose graft_01, 18 May 2017). In brief, the Klein solution was injected (2% lidocaine solution: 0.08% w/v; adrenaline 1 mg/mL solution: 0.1% v/v in 0.9% saline) and left to rest for 8–10 min before starting the liposuction. The cannula and the 20-mL Vac-Lock syringe provided in the kit were used (11G, six holes) to lipoaspirate 20 mL of fat from each donor. Moreover, harvested fat was received and used in experimental protocols, from the University of Verona Laboratory, following the guidelines of the Human Research Approval Committee protocol number 2/2019.

### ED-SVF and HT-SVF productions

The extraction of the SVF was performed following the methodologies already proven in the laboratory, with the enzymatic and mechanic digestion systems. In summary, 20 mL of adipose tissue from each healthy informed donor (n = 3) was divided into two portions. The first portion (10 mL) was processed enzymatically using collagenase type I (Gibco; lot number: 17100017), following the study reported by Busato et al. (2020). The cells extracted using this method were named ED-SVF. The second portion (10 mL) was treated mechanically using a Hy-tissue-SVF kit (Fidia Farmaceutici, Abano Terme, Italy). The method consists of a sterile, single-use double bag, containing an inner filter bag with a mesh size of 120 µm. A volume of 10 mL of lipoaspirate was introduced into the inner porous bag using an upper syringe. The adipose tissue was manually processed for 5 min with the plastic rod located within the microporous bag. The micro-fragmented tissue was then collected through a syringe in the lower part of the outer bag and centrifuged at 1,200 × g for 10 min. The cellular pellet obtained was named HT-SVF. All SVF preparations were obtained freshly from individual donor samples and processed within the same experimental session to minimize inter-sample variability. The use of three biological replicates (n = 3 donors) allowed comparative analysis across treatment groups. No cryopreservation or storage was applied; thus, all samples were used immediately after isolation. Based on current protocols and previous experience in our laboratory, mechanical processing tends to yield SVF products with a lower but more consistent cell count and preserved ECM components, whereas enzymatic digestion results in a higher cellular yield but increased variability depending on the enzyme batch and exposure time.

### ACP preparation and characterization

The ACP used in this study was manufactured by FIDIA Farmaceutici S.P.A. (Abano Terme, Padova, Italy). The ACP formulation has a molecular weight of approximately 200 kDa and undergoes a proprietary auto-crosslinking post-modification process. It was supplied as a sterile, ready-to-use injectable solution and used without further modification. This crosslinking is designed to increase the residence time of the product at the injection site, enhancing the local availability of the bioactive compound during the early regenerative phases. The selection of LMW-HA is based on its known bioactivity in promoting cell migration and proliferation in regenerative contexts. Prior to *in vivo* application, its physicochemical properties (viscosity and molecular weight distribution) were confirmed using gel permeation chromatography and rheological analysis according to the manufacturer’s certificate of analysis. Its selection was based on its known regenerative and scaffold-supportive roles in wound- and bone-healing environments.

### Animal model

Ten-week old, homozygote male nude mice (n = 12) were purchased from Envigo (Milan, Italy). Animals were housed in each cage, with controlled temperature and humidity. They had free access to chops and water. After 1 week of adaptation, animals were housed individually in each cage. To evaluate the potential effect of cellular treatment in bone regeneration, an *in vivo* cranial damage model, known as “Calvaria defect mouse model,” was designed in homozygote nude mice. The animals were randomly divided into four groups: HT-SVF + ACP (n = 3), ED-SVF + ACP (n = 3), ACP (n = 3), and saline solution as the control group (n = 3). Each lipoaspirate (n = 3) was equally used for a single series of experimental groups in order to confront the effect of single-donor lipoaspirates between all the tested conditions.

To perform the cranial defect, animals were first anesthetized with isoflurane (Ugo Basile S.R.L, Varese, Italy). Furthermore, an incision was made using a scalpel, cutting through both dermal layers and the periosteum, to expose the cranial bone. Damage was created bilaterally in the parietal cortex using a mini-drill with a 2-mm-diameter tip.

The created non-trespassing holes (approximately 0.5 mm depth) were filled with 50 µL of ACP mixed with the respective treatment (ED-SVF or HT-SVF), or 50 µL ACP or 50 µL saline. To avoid any leakage of the evaluated treatments, a 2.5-mm-diameter dish of Attiva film (sterile transparent polyurethane adhesive dressing, Svas Biosana S.p.A.) was used to cover the injured cranial bone and then fixed with a drop of tissue glue (3M Vetbond Tissue Adhesive). The superficial skin of the animal was finally sutured with resorbable stitches (737H silk sutures, ETHICON 3-0). In order to ameliorate the animal during postoperative recovery, animals were treated subcutaneously with an analgesic (carprofen, Rimadyl^®^, 1 mL/kg, 1:10; Pfizer, Roma, Italy) and a wide-spectrum antibiotic (enrofloxacin, Baytril^®^, 10 mL/kg, 1:50, Bayer S.p.a., Milano, Italy) every other day, for 5 days.

To ensure humane treatment of the animals, euthanasia was performed using an overdose of sodium pentobarbital, administered intraperitoneally at a dose of 150 mg/kg. This method was chosen for its rapid effectiveness and to minimize animal discomfort. All the procedures involving animals were approved by the Italian NHI, authorization 56DC9.79. All procedures were conducted in full compliance with the ARRIVE guidelines, and all methods were carried out in accordance with relevant guidelines and regulations.

### Immunophenotyping

Cells from ED-SVF and HT-SVF were characterized by flow cytometry. For this purpose, both SVF products were passed through a 45-μm cells strainer to remove cellular aggregates; then, the cell pellet was incubated with 1 mL of erythrocyte lysis buffer 1X (Macs Miltenyi Biotec, Milan, Italy) for 10 min and filtered through a 70-μm cell strainer. Next, cells were collected and counted with trypan blue exclusion assay using a CytoSMART counter (Automated Image-Based Cell Counter, version 1.5.0.16380; CytoSMART Technologies B.V, Eindhoven, Netherlands), and 20,000 cells were washed with PBS (1X) and incubated with a specific monoclonal antibody on ice for 30 min. After incubation, the pellets were centrifuged (5,000 rpm, 7 min) and resuspended in 100 μL of PBS (1X).

The following antibodies were used: CD73 V-450A conjugate (1:5 dilution), CD34 PE-A conjugate (1:20 dilution), and CD105 PerPC-Cy-5-5-A conjugate (1:5 dilution). For cell viability, propidium iodide was used. All antibodies were purchased from BD Biosciences (Becton Dickinson Italy S.P.A., Milano, Italy). Immunophenotyping was performed through a chant II FACS (BD, Becton Dickinson, Milano, Italy).

### Magnetic resonance imaging

The animals were monitored at 5 and 15 days after the surgery through magnetic resonance imaging (MRI) by performing T2_turboRARE_highres analysis (Bruker, Karlsruhe, Germany) equipped with a 7 T (T), 33-cm bore horizontal magnet (Oxford Ltd., Oxford, United Kingdom).

Given that bone is not directly visualized in magnetic resonance, this technique allows to observe the organization and integrity of structures such as the meninges, periosteum, and skin. In addition, the MRI technique provides information regarding the different degree of wound healing by assessing the integrity of tissues adjacent to the point of damage.

### Sample fixation and histological analysis

At 15 dpi, the animals were sacrificed, and the crania were collected for subsequent analysis. The parietal area of the skull was fixed in 4% paraformaldehyde (PFA) for 48 h, decalcified for 24 h using Biodec R (Bio-Optica, Milan, Italy), and then washed with 0.1 M phosphate buffer (pH 7.4). The damage in the right hemisphere of each animal was embedded in OCT and frozen at −20°C. Successively, the samples were cryosectioned at a thickness of 15 µm. For each experimental group, the following histological stainings were performed: hematoxylin/eosin for a morphological evaluation, fibrosis, and inflammatory infiltrates; Alcian blue/Fast red to evaluate the extracellular matrix component formation; Alizarin red/hematoxylin to check the presence of calcium at the site of injury across all the samples. Three slices from each animal were collected for each histological staining. Slides were first washed with PBS 1X for 5 min, stained with Meyer’s hematoxylin (H&E, Bio-Optica, Milan, Italy) for 30 s, washed in running water, stained with 1/10 eosin (H&E, Bio-Optica) for 10 s, and washed with distilled water. Second, slices were washed with PBS 1X, stained with Alcian blue (Merck KGaA, Darmstadt, Germany) for 30 min, washed with distilled water, stained with Fast red solution (Bio-Optica) for 20 min, and washed with distilled water. For the last staining, slices were washed with PBS 1X, stained with Alizarin red (Bio-Optica) for 30 min, washed with distilled water, stained with Meyer’s hematoxylin (H&E, Bio-Optica, Milan, Italy) for 20 min, and washed with distilled water.

Slides were dehydrated with increasing concentrations of alcohol (60, 80, 95, and 100%, each for 10 min), ending with a double wash in xylene for 10 min. Finally, slides were mounted with Entellan (Merck KGaA), dried, and gently cleaned with ethanol. Slides were examined by light microscopy using an Olympus BX-51 microscope (Olympus, Tokyo, Japan) equipped with a JVC DKY-F58 CCD digital camera (Yokohama, Japan).

### Immunofluorescence analysis

Cranial slices were defrosted at room temperature for 10 min and then washed in PBS 1X solution for 10 min and incubated for 30 min in blocking solution (0.25% Triton X-100, 2% bovine serum albumin in PBS 1X). Slices were then incubated overnight at 4°C with a solution containing the selected primary antibody (anti-mouse HLA with human reactivity, Abcam, ab70328; anti-rabbit osteopontin with mouse reactivity, Abcam, ab63856), diluted in blocking solution (1:200). The next day, slices were washed six times for 5 min, each with the blocking solution, and incubated at room temperature under dark condition for 4 h in the secondary antibody solution (anti-mouse ab150113 Abcam, 488; anti-rabbit ab175471 Abcam in 568, respectively), composed by specific secondary antibodies diluted in the blocking solution (1:500). After this step, slices were washed thrice for 5 min, each with the blocking solution, and then washed again thrice for 5 min, each with PBS 1X. Slices were then incubated for 10 min with a solution containing 4,6-diamino-2-phenylindole dihydrochloride (DAPI, Molecular Probes-Thermo Fisher Scientific, 1:2,000) diluted in PBS 1X for the nuclei staining. Finally, slices were washed with PBS 1X, and cover slides were mounted using 1,4-diazabicyclo [2.2.2] octane (DABCO, Sigma-Aldrich).

### Scanning electron microscopy

The damage in the left parietal cortex was processed for scanning electron microscopy (SEM). The samples were post-fixed in 1% osmium tetroxide (OsO4) diluted in 0.1M phosphate buffer for 1 h. Subsequently, the samples were dehydrated by increasing concentrations of ethanol and finally using the critical point dryer (CPD 030, Balzers, Vaduz, Liechtenstein). They were then metallized with gold using an MED 010 coater (Balzers) and were viewed using an FEI XL30 scanning electron microscope (FEI Company, Eindhoven, Netherlands). The images were acquired in transversal cross-section.

## Results

### Characterization of the stromal vascular fraction

**FIGURE 1:**
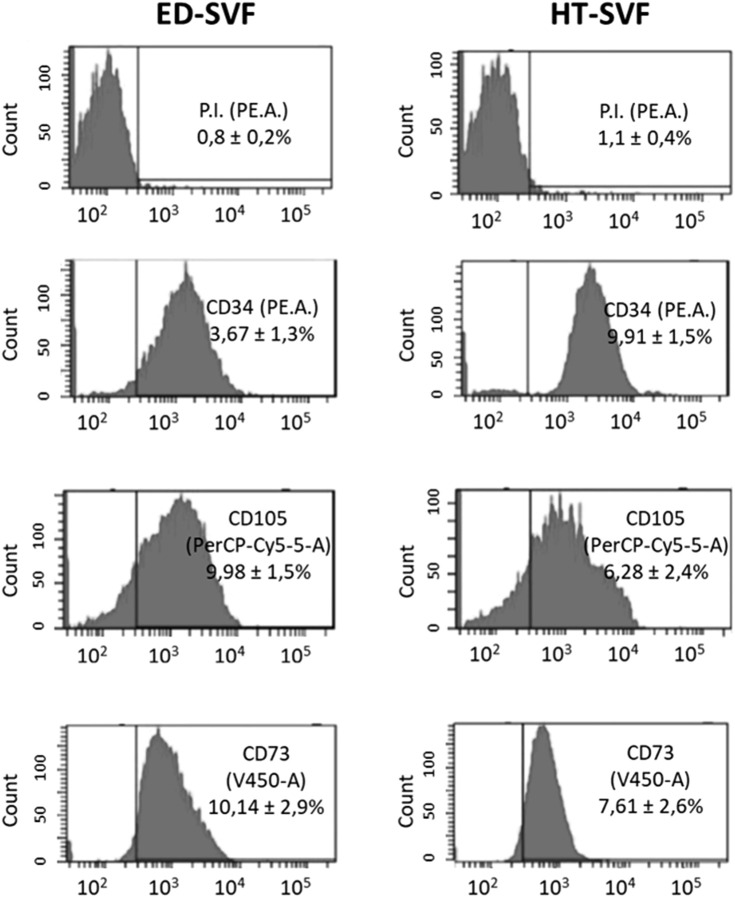
The cell viability and population of ED-SVF and HT-SVF were characterized by flow cytometry by using the P.I., CD34, CD105, and CD73 markers. Data are expressed in percentage of positive cells ±SEM.

FACS analysis displayed high cell viability in both ED and HT-SVF. Moreover, HT-SVF and ED-SVF cytofluorimetric analysis showed that CD34^+^ cell population (endothelial, pericytes, and adipose-derived stem cells) was 9.9% ± 1.5% and 3.7% ± 1.3%, respectively. Additionally, the frequency of cells positive for CD73 and CD105 (mesenchymal stem cells) was analyzed. The percentages of expression in HT-SVF were 7.61% ± 2.59% and 6.28% ± 2.40% for CD73 and CD105, respectively, not thus far from the percentage of expression obtained for the same antibody with ED-SVF (10.14% ± 2.91% and 9.98% ± 1.49%, respectively), as presented in Figure 1.

### Visual evaluation of wound healing

**FIGURE 2:**
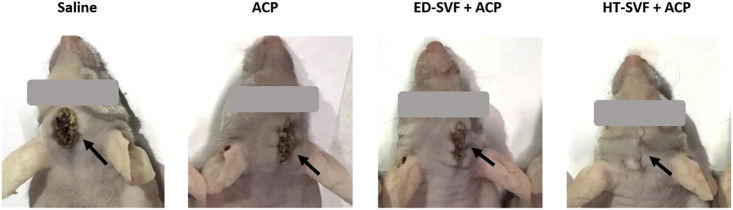
Visual observation of the wound after 15 days post-surgery. The black arrows indicate the wounds. All the experimental groups are presented, including controls (saline and ACP) and treatments (HT-SVF and ED-SVF).

As a follow-up post-surgery procedure, different experimental groups were systematically monitored and evaluated. After the surgery, the degree of wound scarring was assessed at 15 dpi in the different experimental groups through visual evaluation. As shown in Figure 2, the wounds of animals treated with HT-SVF + ACP appeared to be completely healed after 15 days of study, compared to the other experimental groups, which still showed large scars and open wounds.

### *In vivo* monitoring with MRI

After inducing the bone injury, mice were monitored by magnetic resonance at 5 and 15 days after the surgery. The MRI images allowed to control if the defect was performed satisfactorily by supervising that the surrounding structures underlying the cranial bone do not appear compromised.

**FIGURE 3:**
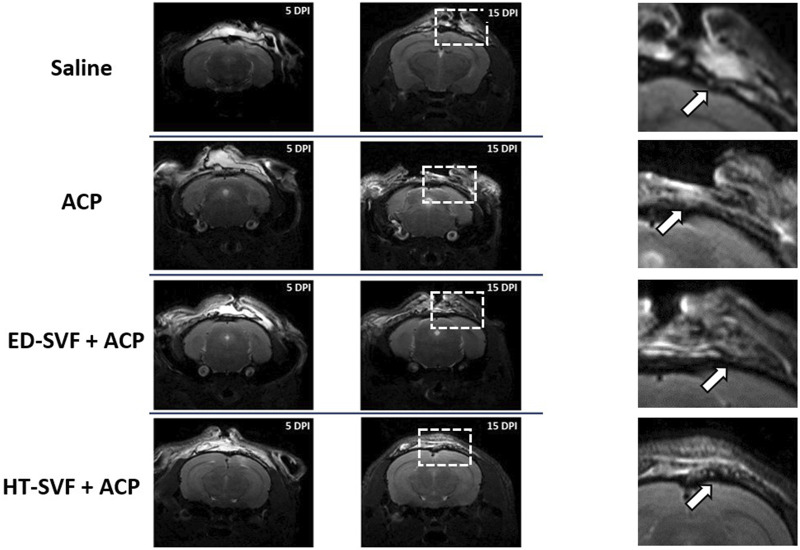
Representative MRI of the wound healing process at 5 and 15 days after the operation. The squares are representative for the magnifications, and the white arrows indicate the injury sites. All the experimental groups are presented, including controls (saline and ACP) and treatments (HT-SVF and ED-SVF).

MRI images (Figure 3) show that treatment with HT-SVF + ACP promotes greater integrity of the skin tissue that covers the cranial bone, unlike all other treatments. In fact, it can be observed how the meninges, periosteum, and skin in the case of treatment with HT-SVF + ACP are structurally more organized and intact, if compared with all the other experimental groups. Moreover, animals that received ED + ACP and ACP showed partial integrity of the observed structures compared to those that received saline treatment. The latter showed a lower integrity of the damaged skin tissues than the other experimental groups.

### Histological analysis

After 15 days of surgery, the treated site of the bone-injured area had been removed to proceed with the different histological analysis. The skulls have been cryo-sectioned and stained with different methodologies that allow us to evaluate different parameters related to the possible regeneration of the damaged bone.

#### Hematoxylin and eosin

**FIGURE 4:**
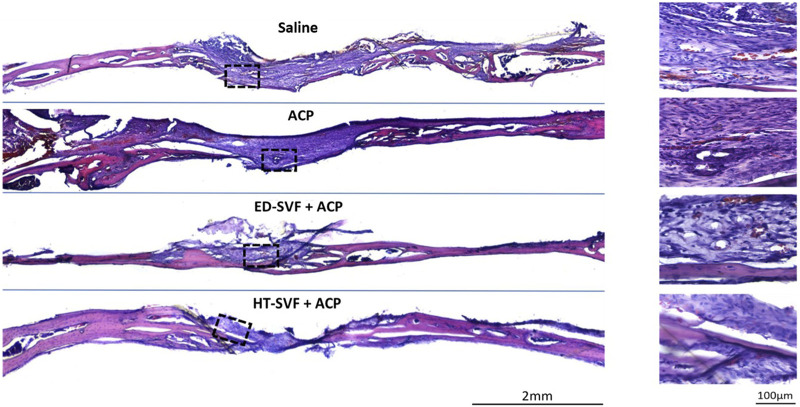
Morphological evaluation with hematoxylin/eosin staining. The squares represent the magnifications in the right column, the black lines represent the initial hole diameter, and the dotted lines represent the newly formed bone area and scar tissue. All the experimental groups are presented, including controls (saline and ACP) and treatments (HT-SVF and ED-SVF).

The hematoxylin and eosin staining analysis had been used on the cross-sectioned calvarial bone in order to study tissue regeneration and their cellular components. As shown in Figure 4, skulls derived from animals treated with saline and ACP have a greater amount of scar material at the site of bone damage than skulls derived from animals treated with HT-SVF + ACP and ED-SVF + ACP, where a lower amount of scar tissue was found. Moreover, the gap in the injured site was reduced, as represented in Figure 4. In fact, the holes of animals that received cellular treatments appeared to have a smaller diameter than those of animals in the controls. However, in Figure 4, on the right, it can also be seen that the bone damage site in HT-SVF + ACP and ED-SVF + ACP treatments had a greater amount of newly formed bone tissue than the bone damage site in saline and ACP controls.

#### Alcian blue and Fast red

**FIGURE 5:**
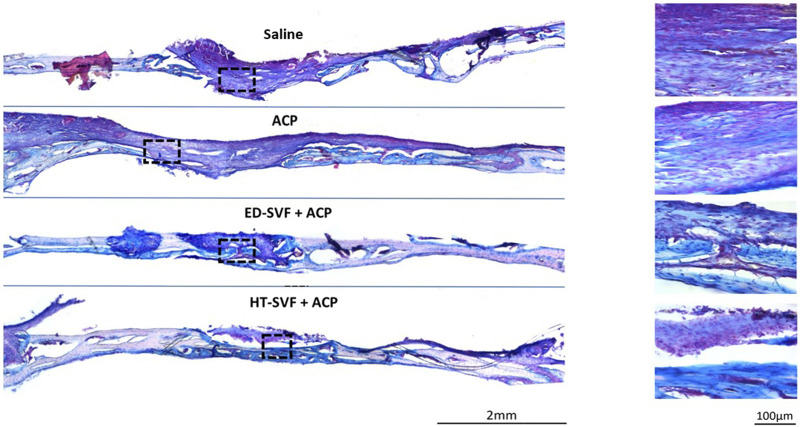
Glycosaminoglycan evaluation with Alcian blue/Fast red staining. The squares represent the magnifications in the right column, the black lines represent the initial hole diameter, and the dotted lines represent the newly formed bone area. All the experimental groups are presented, including controls (saline and ACP) and treatments (HT-SVF and ED-SVF).

As fracture bone healing is associated with transient cartilage formation, we proceeded to investigate the association between cartilage and the bone defects. Therefore, Alcian blue staining identifies the extracellular matrix components, which are essential precursors of bone regeneration, and Fast red stains cell nuclei red. As shown in Figure 5, the skull treated with HT-SVF + ACP shows a greater blue staining intensity degree than that treated with ED-SVF + ACP, which has a high amount of these bone precursor components. In contrast, saline and ACP control groups display poor blue staining at the site of bone injury. The greater blue staining at the site of cranial damage that can be observed in the cellular treatments (HT-SVF + ACP and ED-SVF + ACP) highlights a greater amount of glycosaminoglycan bone precursors, which result in a greater bone regeneration capacity. In particular, HT-SVF + ACP treatment shows a greater thickness of newly formed bone than treatment with ED-SVF + ACP, ACP, and saline controls.

#### Alizarin red and hematoxylin

**FIGURE 6:**
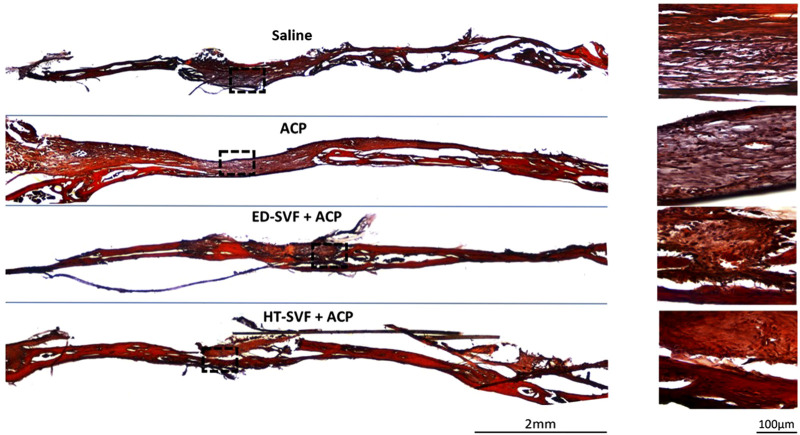
Calcium deposit evaluation with Alizarin red and hematoxylin. The squares represent the magnifications in the right column, the black lines represent the initial hole diameter, and the dotted lines represent the magnification zone of the newly formed area. All the experimental groups are presented, including controls (saline and ACP) and treatments (HT-SVF and ED-SVF).

For further confirmation of bone maturation, Alizarin red has been used to mark calcium deposits, which is the main and essential precursor for the bone regeneration process. As shown in Figure 6, the damage area treated with HT-SVF + ACP shows a greater intensity of red coloration than that treated with ED-SVF + ACP. However, the saline and ACP controls display less staining intensity, as can be seen from the damaged zone magnification in Figure 6. In addition, as observed in the other staining analyses, the HT-SVF + ACP and ED-SVF + ACP treatments show a reduction in the cranial hole diameter. The greater intensity of staining at the cranial damage site that can be observed in cellular treatments is linked to a greater amount of calcium deposits, which is one of the essential components of bone neoformation.

### Immunofluorescence analysis

#### Human cell analysis

**FIGURE 7:**
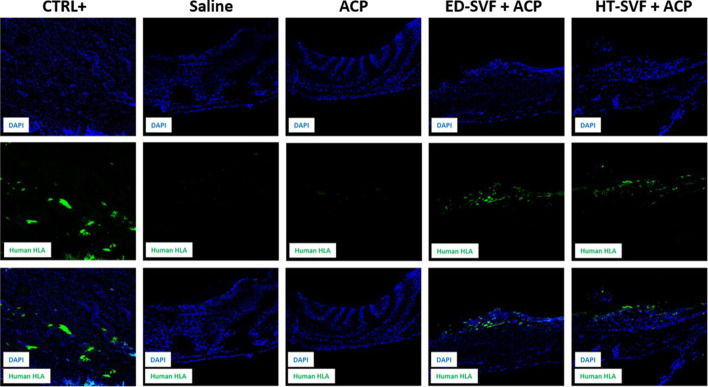
Immunofluorescence analysis of HLA^+^ human cells. The columns consist of different experimental groups (CTRL+, saline, ACP, ED-SVF + ACP, and HT-SVF + ACP). Each row represents different markers, including nuclei marker (DAPI, blue), human leukocyte antigen marker (HLA, green), and composite of markers. All the images were acquired with a ×20 magnification objective.

The immunofluorescence technique was carried out to assess the presence of human cells 15 days after the treatment in the cranial region of injury. Positive control is represented by a human capsular contracture. As shown in Figure 7, the first row in the panel shows the immunofluorescent staining related to DAPI, which marks the cell nuclei in blue; the second row depicts the channel related to the immunofluorescent staining of the human leukocyte antigen (HLA) marker, which is in green; and the third row defines the co-localization of the two channels. As shown in Figure 7, after 15 days, cellular treatments present some positivity of HLA^+^ human cells on the calvarial defect scar surface for the presence of human cells in the HT-SVF + ACP and ED-SVF + ACP treatment groups, whereas there was no positivity in the other experimental groups.

#### New bone formation analysis with osteopontin

**FIGURE 8:**
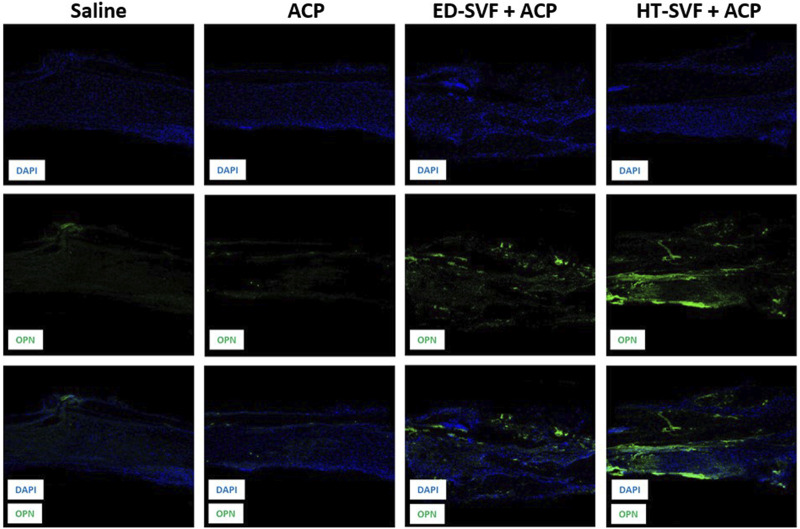
Immunofluorescence analysis of the newly formed bone marker. The columns consist of different experimental groups (CTRL+, saline, ACP, ED-SVF + ACP, and HT-SVF + ACP). Each row represents different markers, including nuclei marker (DAPI, blue), bone neoformation marker (OPN, green), and composite of markers. All the images were acquired with a ×20 magnification objective.

Anti-OPN analysis revealed that the newly formed tissue derived from cellular treatments presents some positivity for the osteopontin marker, which is a neoformation bone marker. As shown in Figure 8, the first row shows the immunofluorescent staining related to DAPI, which marks the cell nuclei in blue; the second row depicts the channel related to the immunofluorescent staining of the osteopontin marker; and the third row defines the co-localization of the two channels. It can be seen from the panel that there is higher positivity of the osteopontin marker in HT-SVF + ACP than in ED-SVF + ACP, whereas weak signal was observed in the other experimental groups (saline and ACP).

### Ultrastructural analysis: scanning electron microscopy

**FIGURE 9:**
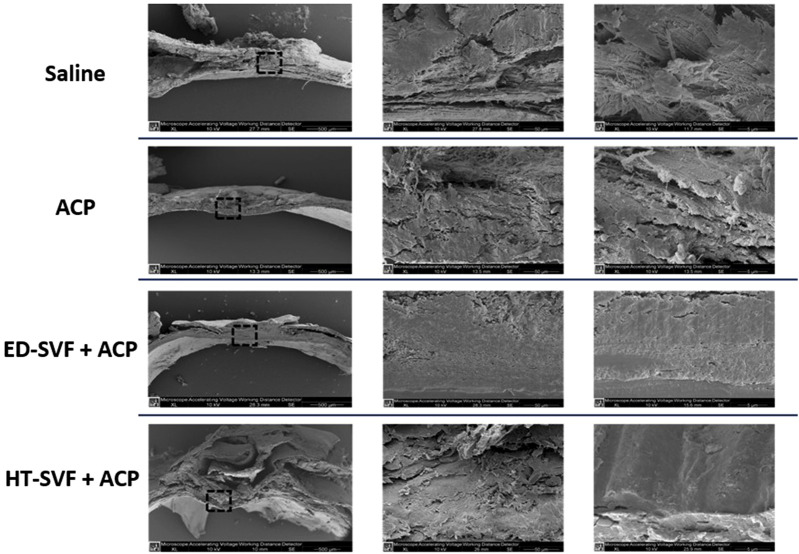
Ultrastructural analysis using scanning electron microscopy (SEM). The squares represent the magnification areas, and the right column represents its further magnification. All the experimental groups are presented, including controls (saline and ACP) and treatments (HT-SVF and ED-SVF).

SEM is a useful technique to analyze the microstructural organization of the sample’s surface. As shown in Figure 9, the newly formed tissue in HT-SVF + ACP and ED-SVF + ACP treatments have a more defined, compact, and organized structure in the cranial damaged region. In contrast, ACP and saline controls show a poorly organized structure, with cracks and breaks in the newly formed material. In addition, both the cellular treatments appear to promote greater bone-like morphology of the newly formed tissue.

## Discussion

Regenerative medicine has profoundly transformed the landscape of bone repair, offering innovative solutions through the integration of stem cell-based therapies and bioactive scaffolds (De Francesco et al., 2023). These approaches address long-standing challenges in treating extensive and complex bone defects, particularly where traditional methods, such as grafting or mechanical fixation, have shown limited efficacy. By leveraging the body’s intrinsic healing mechanisms, cell-based therapies, especially autologous approaches, minimize immunogenic risks and enhance biocompatibility, making them a cornerstone in modern bone regeneration strategies.

In our study, both HT-SVF and ED-SVF demonstrated comparable cell viability and ADSC content, highlighting their potential as regenerative tools. However, HT-SVF emerged as a superior alternative, owing to its ability to preserve the extracellular matrix and secretory factors. This preservation is pivotal, as the extracellular matrix not only provides structural support but also serves as a reservoir for pro-regenerative cytokines, growth factors, and chemokines. These elements collectively create a conducive microenvironment for cellular migration, proliferation, and differentiation, accelerating wound healing and osteogenesis.

The combination of HT-SVF with ACP further amplified its therapeutic efficacy. ACP acts as both a scaffold and a bioactive agent, facilitating the retention and gradual release of regenerative factors while supporting cellular adhesion and tissue integration. Our histological analyses revealed that the HT-SVF + ACP treatment significantly enhanced osteogenesis, as evidenced by the presence of mature bone and enhanced expression of bone precursor markers. These findings underscore the synergy between HT-SVF and ACP in promoting rapid and robust bone regeneration, reducing fibrotic tissue formation, and improving the structural and functional quality of the newly formed bone matrix.

Although ED-SVF + ACP also demonstrated regenerative potential, the differences in outcomes highlight the advantages of mechanical processing. Mechanical digestion methods, as used for HT-SVF, retain micro-fragments of connective tissue and associated vascular niches, which are often disrupted in enzymatic digestion. This retention may explain the superior regenerative outcomes observed with HT-SVF + ACP as these niches are critical for maintaining cellular viability and functionality. Although this study focused on calvarial bone regeneration, the biological features of SVF, including its multi-lineage differentiation capacity and secretory profile, make it a promising candidate for applications in other tissues. The preservation of stromal architecture in HT-SVF further supports its potential translational use across various regenerative medicine fields.

Despite the promising results, there are limitations to be considered. The reliance on a murine model introduces challenges in extrapolating findings to human clinical scenarios. Murine physiology, particularly in bone remodeling and healing dynamics, differs significantly from that of humans. Additionally, the relatively short follow-up period in this study limits the ability to assess long-term outcomes, such as the durability and integration of the regenerated bone under physiological stress. It is important to acknowledge that the present study is limited to the use of a murine calvarial defect model, which, although valuable for initial proof-of-concept data, does not fully replicate the complex biomechanical and physiological environment of human bone tissue. Moreover, the 15-day follow-up period, although sufficient to evaluate early stages of healing and bone matrix formation, precludes assessment of long-term outcomes such as structural remodeling, integration, and mechanical strength. Future studies should consider larger animal models and extended monitoring periods to validate the translational potential of HT-SVF + ACP therapy.

Future research should address these gaps by extending the duration of studies and incorporating larger animal models that better mimic human bone physiology. Translational studies focusing on the scalability, safety, and efficacy of HT-SVF + ACP in clinical settings are crucial. Moreover, exploring the potential of combining HT-SVF with other bioactive molecules or advanced scaffold designs could further enhance its regenerative capabilities. Investigating its application in more complex defect models, such as those involving load-bearing bones or multi-tissue interfaces, would also provide valuable insights into its clinical utility.

In conclusion, the findings from this study establish HT-SVF as a transformative approach in bone regenerative medicine. Its cost-effectiveness, simplicity, and superior regenerative outcomes position it as a viable alternative to enzymatic methods. When combined with bioactive scaffolds like ACP, HT-SVF holds the potential to address a wide range of clinical challenges in bone repair, paving the way for innovative, patient-specific therapies that restore both form and function in severe and complex bone injuries.

## Conclusion

In this study, we demonstrate that HT-SVF significantly enhances the healing process of bone defects, outperforming conventional methods. Cranial bone regeneration was characterized by early connective tissue formation in the cortical bone region, followed by accelerated remodeling and maturation. The rapid healing observed with HT-SVF + ACP can be attributed to its enriched regenerative compounds and the synergistic effect of ACP, which together provide a robust scaffold for bone growth.

Our findings underscore the pivotal role of SVF, not only in delivering stem cells essential for bone repair but also in leveraging the trophic effects of the extracellular matrix and associated regenerative niches preserved through mechanical purification. Moreover, HT-SVF presents a simplified, cost-effective alternative to enzymatic methods, making it a viable option for clinical application.

In conclusion, these results establish the efficacy of SVF supplementation as a transformative approach for severe bone injuries. The insights gained pave the way for combining SVF with other bioactive molecules, such as hyaluronic acid, to develop innovative, highly effective strategies for enhancing bone regeneration and repair in complex clinical scenarios.

## Data Availability

The raw data supporting the conclusions of this article will be made available by the authors, without undue reservation.
